# 

**Published:** 1900-04

**Authors:** 


*Fifty-Fifth Year.*

VOL. LV.
Whole Number,
DCXLI.

APRIL, 1900.

New Series.

VOL. XXXIX.

No. 9.

LIBRARY
Indexed
30

### BUFFALO MEDICAL JOURNAL

**Established 1845 by AUSTIN FLINT, M. D.**

**EDITOR:**

WILLIAM WARREN POTTER, M. D.

**ASSISTANT EDITORS:**

WILLIAM C. KRAUSS, M. D. NELSON W. WILSON, M. D.

**ASSOCIATE EDITORS:**

JAMES WRIGHT PUTNAM, M. D. ERNEST WENDE, M. D.
JOHN PARMENTER, M. D. ALVIN A. HUBBELL, M. D.
JOHN A. MILLER, Ph. D. MAUD J. FRYE, M. D.

LIBRARY
SURGEON GENERAL'S OFFICE
MAR. 21. 1900

**EUROPEAN AGENCY**, J. B. BAILLIERE & FILS, 19 Rue Hautefeuille, Paris.
*Subscription, if paid in advance, $2.00 ; otherwise, $2.50. Foreign subscription, $2.50*
Copyright 1900, by William Warren Potter, M. D.
Entered at the Post Office at Buffalo, N. Y., as second-class mail matter.

A. T. BROWN PRINTING HOUSE, BUFFALO, N. Y.

**Table of Contents on Page V.**

